# Evaluation of Coated Biochar as an Intestinal Binding Agent for Skatole and Indole in Male Intact Finishing Pigs

**DOI:** 10.3390/ani11030760

**Published:** 2021-03-10

**Authors:** Dana Carina Schubert, Bussarakam Chuppava, Franziska Witte, Nino Terjung, Christian Visscher

**Affiliations:** 1 Institute for Animal Nutrition, University of Veterinary Medicine Hannover, Foundation, Bischofsholer Damm 15, D-30173 Hanover, Germany; Bussarakam.Chuppava@tiho-hannover.de (B.C.); Christian.Visscher@tiho-hannover.de (C.V.); 2 German Institute for Food Technologies (DIL e.V), Quakenbrück, Prof.-von-Klitzing-Straße 7, D-49610 Quakenbrück, Germany; f.witte@dil-ev.de (F.W.); n.terjung@dil-ev.de (N.T.)

**Keywords:** skatole, indole, boar, boar taint, piglet castration, biochar, plantcoal

## Abstract

**Simple Summary:**

Public awareness of animal welfare in livestock farming continues to grow all over the world, especially in Central Europe as well as in Australia and Northern America. Consequently, the ban on piglet castration without anaesthesia comes into force in Germany and France in 2021 and other European countries such as Norway, the Netherlands, Switzerland and Sweden have taken legislative actions against piglet castration without pain relief earlier. Hence, alternatives have to be established that prevent the occurrence of boar taint in male pigs. The aim of the present study was to examine the effect of dietary biochar on boar taint compounds (skatole and indole) in faeces and plasma as well as on performance parameters. The biochar was added to the feed whether two or four weeks before slaughter. Animals that received biochar during the last two weeks before slaughter had lower skatole concentrations in faeces at the end of the trial than at the beginning, whereas these results could not be confirmed when animals received biochar for four weeks. Nevertheless, the faeces was drier when animals were fed biochar and performance was not affected. Results indicate that more research is necessary to better understand the nonspecific adsorption capacity of biochar.

**Abstract:**

The ban on piglet castration without anaesthesia poses a challenge for the meat industry since alternatives ensuring the production of flawless pork have to be established. The aim of this study was to evaluate the effects of biochar on skatole and indole concentration in faeces and plasma on a small scale in finishing boars to prove whether biochar was suitable for use in commercial pork production. Moreover, it was investigated whether biochar affects faecal properties or the performance. For a four-week trial period, 54 boars (bodyweight 97.2 ± 6.88 kg) were divided into three groups. The control (BC0) received no dietary biochar, one group received a diet containing 4% coated biochar (corresponding to 2% pure biochar) for the final two experimental weeks (BC2), and another group for the entire four weeks (BC4), respectively, prior to slaughter. Skatole and indole concentrations were measured in faeces and plasma at the beginning, in the middle and at the end of the trial. Mean skatole concentrations did not differ between groups, but in BC2 faecal skatole was significantly decreased at day 26, whereas in BC4 initial and final faecal skatole levels did not differ. At day 15 and 26, the faecal dry matter content was significantly higher in pigs fed the biochar diet (*p* < 0.05).

## 1. Introduction

The awareness of animal welfare-friendly livestock farming is growing nearly worldwide but especially in Central Europe, Northern America and Australia. Consequently, increasing numbers of countries are banning piglet castration without anaesthesia or pain relief [1,2]. Hence, piglet producers as well as pig farmers have been faced with the challenge of implementing financially viable alternatives to piglet castration without anaesthesia that prevent the development of boar taint in pork [3].

The term boar taint describes odour and taste deviations in the carcass, which are mainly caused by androstenone (5α-androst-16-ene-3-one) and skatole (3-methyl indole) [4,5,6,7]. Partially, indole also causes the occurrence of boar taint [8,9]. Differences between the two substances androstenone and skatole exist not only in terms of their physiology and metabolism but also in their perceptibility and odour quality. Androstenone cannot be perceived by about 30% of consumers and is described as urine-like, whereas skatole is recognised by almost all consumers and is characterised as a faecal-like odour [6,10,11].

Androstenone functions as a sex pheromone. Its formation in the testes is strongly correlated to sexual steroids such as testosterone, so that by reducing androstenone production, the positive effects of the anabolic testicular hormones on the carcass are usually lost. Skatole and indole are formed by microbial degradation of the amino acid tryptophan (TRP) in the colon [12,13]. This process occurs not only in boars but also in monogastric species in general [13]. However, particularly high concentrations of skatole are found in boars, as sex steroids are presumed to inhibit the hepatic metabolism and clearance of skatole [14].

There are basically three alternatives to surgical castration without anaesthesia, namely surgical castration under (general) anaesthesia, immunocastration and fattening of entire males. Even though anaesthesia prevents pain during surgery, animals still suffer from postoperative pain. The injection pain of immunocastration is negligible; however, this practice has not been able to gain acceptance in many areas due to reservations from consumers. Fattening of intact boars offers the advantage of leaner meat and higher growth rates due to anabolic testicular hormones but involves, among these alternatives, the highest risk of elevated boar taint levels [15].

Nevertheless, with regard to the physiology of skatole formation and skatole metabolism, there are various nutritive approaches to reducing skatole concentrations in boars [16,17,18]. As successfully shown by several studies, changes in TRP availability consecutively changed skatole concentrations [19,20,21]. Feeding dietary protein with a low precaecal digestibility as well as the induction of higher apoptosis rates in the intestine increased TRP availability in the hindgut and thus skatole formation [19,20,21,22]. Although it was not possible to reduce skatole formation through lower TRP contents in the diet, the reduction in apoptosis rates successfully decreased skatole concentrations in the hindgut [22,23]. The latter has been achieved by feeding components such as raw potato starch, which are fermented in the large intestine to butyrate [22]. Raw potato starch also offers the advantage of replacing TRP as an energy source for the bacteria in the colon. Subsequently, the TRP is not degraded to skatole but used for microbial protein synthesis. Similar effects were shown for non-starch polysaccharides (NSP), which are precaecally only partially digestible or even indigestible, e.g., inulin [24,25,26]. Feeding trials revealed a minimum dietary level of 20% for potato starch and of 5–10% for inulin (either pure or as a component of the chicory root or Jerusalem artichoke) to achieve a measurable skatole reduction in the fat tissue [17]. Another way of influencing microbial activity in the chyme was shown in vitro by Jensen et al. [12] who demonstrated that the ratio of skatole to indole production changes depending on the prevailing pH values. At pH values in the range of 8.0, the ratio shifts in favour of indole, but at pH 5.0, it shifts in favour of skatole. Except for reducing skatole formation, accumulation of skatole in fatty tissue can also be minimised by enhancing the hepatic metabolism of skatole [16,27,28].

The use of adsorbent materials to achieve an increased faecal excretion by intestinally binding boar taint compounds yielded contradictory results [29,30,31]. In finishing boars, adding 0.5% zeolite (which corresponds to 0.45% clinoptilolite) to the diet significantly reduced skatole concentrations in adipose tissue [29], whereas dietary levels of 1% clinoptilolite were not successful in reducing skatole concentrations [30]. The authors Jen and Squires [32] investigated the capacity for binding skatole and androstenone of various adsorbents in vitro. Based on these results, they selected activated carbon and tween-60 for in vivo application in Yorkshire boars (initial bodyweight 119.8 kg) [31]. Feeding 5.0% activated carbon or 5.0% tween-60 for 28 days resulted in decreased androstenone levels in the plasma and back fat of the boars, whereas no effect on skatole concentrations in the plasma was detectable for either adsorbents and in the adipose tissue, the concentrations in the tween-60 group increased after 28 days. The authors assumed that the missing effects on skatole were related to the fact that skatole levels were generally very low in all groups (skatole level <200 ng/g in adipose tissue).

Biochar is, similar to activated carbon, produced by pyrolysis of biomasses. Both have a large and porous surface area in common, enabling them to adsorb various substances [33]. In contrast to biochar, production of activated carbon requires thermal or chemical activation, which leads to an enlargement of porosity [34]. For this reason, the production of biochar is more economical and therefore more suitable for large-scale use in pork production.

Furthermore, the use of biochar as a feed component was shown to improve animal health, increase nutrient intake efficiency and thus productivity in livestock. Additionally, due to its unspecific absorption capacity, charcoal is a widespread treatment for oral intoxications and diarrheal infections [35].

The aim of the present study was to examine the effects of coated biochar on skatole and indole concentrations in faeces and plasma to prove whether biochar could be applied either two or four weeks prior to slaughter to act as an intestinal binding agent for skatole and indole. Levels might be fostered in practice due to stress which can occur as a consequence of regrouping the animals especially in the last weeks before slaughter. Furthermore, it was evaluated if adding coated biochar to the compound feed of finishing boars affects faecal properties or performance parameters.

## 2. Materials and Methods

The experiments were conducted in accordance with German regulations and approved by the Ethics Committee of Lower Saxony for the Care and Use of Laboratory Animals (LAVES: Niedersächsisches Landesamt für Verbraucherschutz und Lebensmittelsicherheit; reference: 33.8-42505-05-18A334).

### 2.1. Animals and Housing

The study was conducted in a total of 54 finishing boars (*N* = 54) with an initial body weight of 97.2 ± 6.88 kg. The animals were divided into three consecutive trials (T1, T2, T3) of 18 boars each (*n* = 18 per trial). The boars originated from one commercial pig farm in Lower Saxony, Germany and were cross breeds of BHZP Pietrain (sire line) and Danish landrace (dam line). All 54 boars were descended from a pool of five sires with similar heritability regarding androstenone and skatole.

The animals were housed individually in 1 × 3 m^2^ boxes with a solid floor, equipped with a 1 m long metal trough and nipple drinkers. Boxes were equipped with environmental enrichment materials and peepholes to allow nose-nose contact between the boars. Visual contact to the other pigs was provided through metal bars in the doors at the front of the boxes. The lighting period was set from 07:00 to 19:00 h.

### 2.2. Diets and Feeding Concept

Water was provided ad-libitum by a nipple drinker and feed was provided restrictively according to BHZP recommendations for this crossbreed [36]. Feed was offered twice daily in equal shares at 08:30 and 16:30 h, respectively. Two different granulated complete feeds (variant 1 and variant 2) were used as the basis for the experimental diets control feed (CON) and biochar containing feed (BCF). The two variants consisted of barley, wheat, triticale, rye, wheat bran and soy extraction meal, inter alia (Table 1). The control feed (CON) corresponded to a standard feed for finishing pigs and was based on variant 1, whereas variant 2 was used to create BCF, which contained 2% (*w*/*w*) biochar.

On the basis of past experiments with pure biochar, which were conducted in piglets as they show particularly high skatole formation [37], it was decided to coat the biochar with a rapeseed fat (1:1 *w*/*w*) as the pure biochar did not show a reliable reduction neither of skatole nor indole (data not published). From this strategy, the authors hoped that the fat would be digested during its passage through the small intestine by endogenous enzymes and that the biochar would be free of fat and the pores unloaded when entering the hindgut. To achieve a concentration of 2% biochar in the experimental feed, 4% of the biochar-fat mix was added to the feed. In order to maximize the comparability of the two diets, pure vegetable fat was added to the control feed (2% *w*/*w*). To ensure that the diets (CON and BCF) were approximately iso-energetic and iso-nitrogenous, variant 2 was a slightly concentrated form of variant 1. The analytical composition of the diets is listed in Table 2.

### 2.3. Experimental Design and Sampling

**Figure 1:**
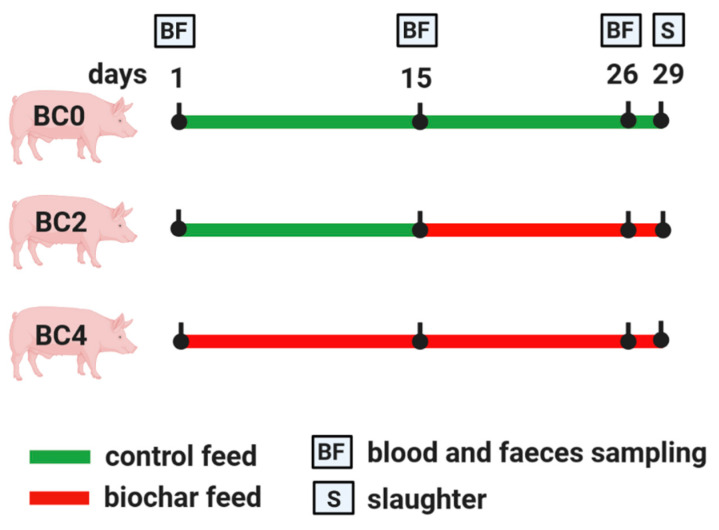
Overview regarding experimental procedure and sampling collection.

After a five-day adaption period, during which all animals received CON, the animals were divided into three groups. The experimental period lasted a total of four weeks. The control group (BC0) received CON over the entire experimental period. The second group (BC2) received CON during the first two weeks and BCF for the last two weeks of the trial, whereas the third group (BC4) received the BCF over the entire experimental period of four weeks. At the end of each trial, all animals were slaughtered. Body weight was measured weekly at the beginning of each experimental week and one day before slaughter. Blood samples and faecal samples for determining skatole (S) and indole (I) concentrations were collected at day 1, 15 and 26. Blood samples were taken by puncturing of the anterior vena cava using plasma monovettes (S-Monovette^®^ K3 EDTA, 9 mL, Sarstedt AG & Co., Nümbrecht, Germany). Faecal samples for measuring dry matter content and pH-value of the faeces were collected at day 1, 8, 15, 22 and 26. Furthermore, samples from subcutaneous fat were obtained from the neck region of each animal as described by Mörlein et al. [38] one day after slaughter. Figure 1 shows the experimental procedure.

### 2.4. Analytical Methods

All analyses were performed in duplicate. The feed was analysed by standard procedures in accordance with official methods of the VDLUFA [39]. Dry matter was determined by drying samples to weight constancy at 103 °C. Crude ash was determined by means of incineration in the muffle furnace at 600 °C for 6 h. The DUMAS combustion method (Vario Max^®^, Elementar, Analysen-Systeme GmbH, Hanau, Germany) was used to analyse the total nitrogen content. Total N was multiplied by a constant factor of 6.25 to calculate the crude protein content. The crude fat content was analysed after acid digestion in the Soxhlet apparatus. The crude fibre content was determined after washing the samples in diluted acids and alkalis. To detect the starch content, an enzymatic determination (UV-method, R-Biopharm AG, Darmstadt, Germany) was applied. Atomic absorption spectrometry (Unicam Solaar 116, Thermo Fischer Scientific GmbH, Dreieich, Germany) was used to detect minerals.

The concentration of S and I in faeces as well as in plasma was measured by means of high-performance liquid chromatography (HPLC LC10, Shimadzu Inc., Kyoto, Japan) with fluorescence detection (RF10 AXL, Shimadzu Inc., Kyoto, Japan). The detection of S and I in plasma was performed as described by Claus et al. [40], the detection in faeces was carried out in accordance with the method described by Dehnhard et al. [41], with the difference that a column produced by Waters (Oasis HLB 6cc Vac Cartridge 200 mg, Waters Inc., Milford, MA USA) was used for purification of the samples.

Using a digital probe (pH 526, Wissenschaftlich-Technische-Werkstätten, Weilheim, Germany; Electrode BlueLine, Schott Instruments GmbH, Mainz, Germany), the pH value in faeces was determined after adding approx. 5 g of sample material to 10 g of distilled water and then homogenising the samples.

The analysis of back fat regarding S, I and androstenone levels was performed in the German Institute for Food Technologies (DIL, Deutsches Institut für Lebensmitteltechnik e. V., Quakenbrück, Germany) by means of UPLC-MS/MS (ACQUITY-UPLC-System, Waters Inc.; API4000, AB Sciex GmbH, Darmstadt, Germany) in accordance with Gibis et al. [42].

### 2.5. Statistical Analysis

For statistical analysis, the Statistical Analysis System for Windows SAS^®^ Enterprise Guide^®^, version 7.1 (SAS Institute Inc., Cary, NC, USA) was used. Mean values and the standard deviation were calculated for all parameters. All data or the residual distribution were tested for normal distribution. For parameters S as well as I concentration in faeces, ADWG and G:F, a two-way analysis of variance (ANOVA) with treatment (BC0, BC2, BC4) and trial (T1, T2, T3) as independent factors was conducted. In the case of plasma concentrations of S and I, the Kruskal–Wallis Test and consecutively the Wilcoxon Test were performed as the corresponding data were mostly not normally distributed. Furthermore, the t-Test or signed-ranked-test, depending on whether normal distribution was present, were performed to compare dependent samples of the parameters S and I content in faeces and plasma. If the S and I values were below the detection limit (faeces: 10 µg/g TS, plasma: 10 ng/dL), half the detection limit was taken as the corresponding value. In terms of faecal pH and DM, outliers were removed from the statistics if these differed from the mean by more than three standard deviations. Differences with a significant level of *p* < 0.05 were taken to be statistically significant.

## 3. Results

### 3.1. Skatole and Indole Concentrations in Faeces and Plasma

The results of S and I measurements in faeces and plasma are shown in the supplementary material Tables S1–S4. The initial faecal concentrations were 120 ± 101 µg/g DM (S) and 58.3 ± 32.7 µg/g DM (I) and did not differ between treatments or trials, whereas the sum of S and I (S + I) in faeces was significantly lower in BC4 than in BC2 at day 1 (BC0: 192 ^ab^ ± 119; BC2: 227 ^a^ ± 112; BC4: 131 ^b^ ± 76.9 µg/g DM). No further significances between the treatments regarding faecal boar taint compounds were detected (*p* > 0.05).

When comparing the trials, faecal S was significantly higher at day 15 in T1 than in T2 and T3 (T1: 288 ^a^ ± 130; T2: 166 ^b^ ± 63.6; T3. 144 ^b^ ± 62.4 µg/g DM) and at day 26 higher in T1 than in T3 (T1: 155 ^a^ ± 75.9; T2: 130 ^ab^ ± 59.3; T3: 97.5 ^b^ ± 54.2 µg/g DM). The same applied for faecal S + I at day 15 (T1: 369 ^a^ ± 131; T2: 220 ^b^ ± 72.4; T3: 207 ^b^ ± 54.4 µg/g DM), whereas the S + I did not differ at day 26. Faecal I differed between the trials at day 1 (T1: 70.1 ^a^ ± 36.2; T2: 63.0 ^ab^ ± 27.1; T3: 41.7 ^b^ ± 28.5 µg/g DM) but not at day 15 or day 26.

For further evaluation, the dependent samples (a total of three measurements each for the S and I contents in faeces and plasma per animal) were evaluated by means of a t-test or sign-rank test. For this purpose, the differences between day 1 and day 15, day 15 and day 26 as well as day 1 and day 26 were calculated. The means of the differences are shown in Tables S2 and S4. In general, faecal S levels increased from day 1 to day 15, thereafter decreasing up to day 26 (*p* < 0.05), but the values at day 26 were not significantly different from day 1 (*p* = 0.887). Nevertheless, in BC2 and T3, respectively, the increase from day 1 to day 15 was not significant (*p* > 0.05). With regard to faecal I, no significant increase was detected for the single treatments (*p* > 0.05). However, in BC0 and BC2 there was a significant decrease from day 15 to day 26 (*p* < 0.05) and from day 1 to day 26 (only BC2, *p* = 0.014). Faecal S + I levels followed the same pattern as the S levels with the difference that in BC2 there was a significant decrease from day 1 to day 26 (*p* = 0.020).

The initial mean plasma concentrations of S (16.9 ± 30.6 ng/dL), I (19.3 ± 32.1 ng/dL) and S + I (34.9 ± 60.3 ng/dL) did not differ between the treatments or trials (*p* > 0.05). In addition, during the rest of the experiment no significant differences between the treatments regarding mean plasma concentrations of S, I and S + I were observed, whereas at day 26 S in plasma was higher in T2 than in T1 and T3 (T1: 11.0^b^ ± 7.79; T2: 43.1^a^ ± 87.5; T3: 12.7 ^b^ ± 18.0 ng/dL) and at day 15 I in plasma was higher in T3 than in T1 and T2 (T1: 17.6 ^b^ ± 6.82; T2: 17.7 ^b^ ± 5.90; T3: 40.9 ^a^ ± 48.3 ng/dL).

**Figure 2:**
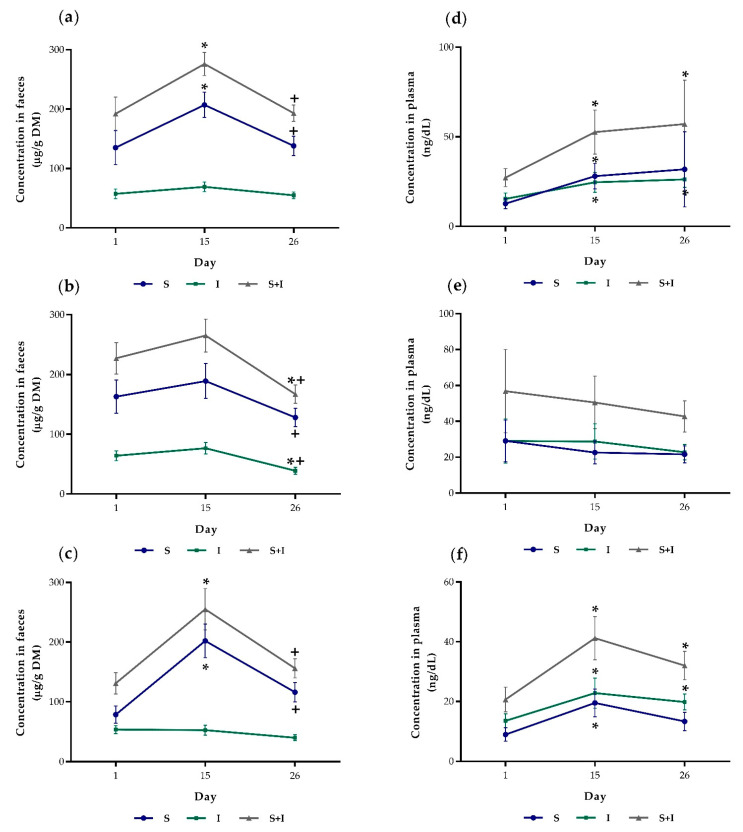
Skatole (S), indole (I) and sum of skatole and indole (S + I), concentrations (mean ± S.E.M.) in faeces of BC0 (**a**), BC2 (**b**) and BC4 (**c**) as well as plasma of BC0 (**d**), BC2 (**e**) and BC4 (**f**), respectively. * Indicates values are significantly different from values at day 1. ^+^ Indicates values are significantly different from values at day 15. DM, dry matter. SEM, standard error of the mean.

Similar to faecal S, also the plasma concentration of S increased from day 1 to day 15 in BC0 (*p* = 0.021) and BC4 (*p* = 0.001) but not in BC 2 (*p* = 0.804). Furthermore, in BC0 and BC4, plasma I was significantly increased at both day 15 and day 26 compared to day 1 (*p* < 0.05). Plasma S + I increased from day 1 to day 26 in BC0 (*p* = 0.043) and BC4 (*p* = 0.008). Figure 2 graphically shows the course individual of S, I and S + I concentrations in faeces and plasma, for each treatment (BC0, BC2 and BC4, respectively).

### 3.2. Dry Matter Content and pH-Values in Faeces

The faecal dry matter content (DM_f_) and the faecal pH (pH_f_) are shown in dependence on feed affiliation (Table 3), which is defined as diet fed the previous day. During the adaption phase before the start of the study, all pigs were fed CON. Therefore, at day 1 no values for BCF are available. Furthermore, this methodological approach had the effect of changing the sample size after day 15, as BC2 was initially assigned to CON, and after day 15, to BCF.

The DM_f_ was significantly higher in BCF than in CON at day 15 (CON: 283 ^b^ ± 23.4; BCF: 301 ^a^ ± 29.2 g/kg) and day 26 (CON: 286 ^b^ ± 27.4; BCF: 305 ^a^ ± 31.7 g/kg), whereas at day 8 and day 22 the means were not significantly different. The mean pH_f_ was in the range of 6.77 to 7.03 and did not differ between CON and BCF. Additionally, the *t*-test revealed that both, DM_f_ and pH_f_, were significantly higher at day 26 compared to day 1 (*p* < 0.05, *N* = 52).

### 3.3. Performance Parameters

The results of the performance parameters are shown in Table 4. The BW at day 1 was 97.2 ± 6.88 kg and neither differed between groups nor between trials. At the day before slaughter, the animals weighed an average of 120 ± 6.28 kg. In trial 1, the average daily weight gain (ADWG, given in grams per day, g/d) was significantly higher than in trials 2 and 3 (T1: 925 ^a^ ± 79.0; T2: 798 ^b^ ± 128; T3: 788 ^b^ ± 136 g/d) but did not differ between treatments (BC0: 806 ± 159; BC2: 837 ± 126; BC4: 865 ± 102 g/d). The animals received 34–36 MJ ME daily depending on the average body weight of the trial in according to BHZP recommendations for this crossbreed [36]. Usually, the animals ate the feed completely. Nevertheless, occasional feed residues were taken into account in the calculation of feed conversion.

### 3.4. Correlations

Correlations were calculated between skatole and indole in faeces and plasma, as well as between faecal skatole and indole concentrations and pH_f_. Furthermore, correlation tests were conducted to evaluate the associations between boar taint compounds at day 1 and day 26 and between plasma concentrations at day 26 and concentrations in back fat (samples taken after slaughter).

The supplementary material Table S5 shows Spearman’s rank correlations coefficients and the corresponding *p*-value. A footnote indicates if the Pearson’s coefficient was shown when both parameters were normally distributed.

Considering the animals independently of the treatment (*N* = 54), positive correlations were found between skatole concentrations in faeces and plasma at day 1 and day 15 (*p* < 0.05), whereas skatole concentrations in faeces and plasma at day 26 only tended to correlate. With regard to indole, concentrations in faeces and plasma did not correlate at any time point. Furthermore, skatole concentrations in faeces as well as in plasma at day 1 correlated to corresponding concentrations at day 26. The indole values in plasma at day 1 also correlated with the values at day 26, but in faeces, there was no correlation (*p* > 0.05). Additionally, strong positive correlations were found between plasma skatole (day 26) and skatole in back fat and plasma indole (day 26) and indole in back fat (*p* < 0.0001). No correlations were found between the levels of skatole and indole or androstenone in back fat (*p* > 0.05), whereas back fat indole and androstenone correlated (*p* = 0.011). Correlations were also found between faecal pH and indole concentrations at day 1 and day 15 but not at day 26. Faecal skatole did not correlate to pH_f_. There were no correlations between performance parameters (BW, ADWG, G:F) and skatole levels.

## 4. Discussion

The abandonment of piglet castration without anaesthesia is increasingly recognized worldwide. Countries such as the United Kingdom, Ireland, New Zealand and Australia have already dispensed with surgical castration for years [43] and in large parts of Europe, castration without anaesthesia or at least without pain relief is meanwhile prohibited [1,2]. Therefore, animal welfare-friendly alternatives must be implemented instead to prevent the occurrence of boar taint in the carcasses. The aim of the present study was to evaluate whether biochar could be suitable for use in commercial pork production to reduce the occurrence of boar taint as well as to contribute to animal welfare in general. The biochar used in the present study was made from oak and was selected on the basis of preliminary experiments on a wide range of different biochars. The investigations showed that skatole and indole bind well to the biochar in vitro (data not published). Prior to this study, it had also been checked whether skatole and indole, once bound to biochar in the faeces, would be detected in the HPLC analysis. The results indicated that only unbound (“free”) skatole and indole contents were measured.

The study conducted in 54 finishing boars was divided into three consecutive trials (T1, T2, T3). No animal losses occurred during the trials. Nevertheless, in T1, one animal from BC4 was removed from the statistical evaluation of the performance parameters on account of developing a stomach ulcer.

The concentrations of skatole and indole in faeces and plasma did not differ between the treatments (*p* > 0.05). Only faecal S + I at day 1 was significantly lower in BC 4 than in BC2 (BC0: 192 ^ab^ ± 119; BC2: 227 ^a^ ± 112; BC4: 131 ^b^ ± 76.9 µg/g DM). However, this was the initial value and cannot be attributed to the biochar treatment. Since the comparison of the mean values did not allow any statement regarding the effect of coated biochar, paired (dependent) samples were additionally used to check whether the concentrations within a treatment differed significantly between the measuring times (day 1, day 15 and day 26, respectively). Both in faeces and plasma S and S + I increased from day 1 to day 15 in BC0 and BC4. In BC2 no significant increase in plasma and faecal S and I levels were detectable, but the faecal concentrations were lower at day 26 than at day 1 (Figure 2). The divergent variations in skatole and indole concentrations between the experimental groups can only with difficulty be attributed to the biochar treatment as BC2 differed from BC0 already between measurements taken at day 1 and day 15, but the animals in this group did not receive biochar during this period. Therefore, other factors are accountable for the differences. This can also be deduced from the fact that the mean values of S, I and S + I differed more often between the three trials than between the treatments (Tables S1–S4).

Compared to Rasmussen et al. [44] and Hansen et al. [45] who reported mean skatole levels of 410 ng/dL and 243 ng/dL, respectively, in plasma of finishing boars (Duroc × (Danish Landrace × Yorkshire)), the skatole levels found in the present study were quite low as only one animal had a skatole level of 410 ng/dL in plasma, whereas the rest of the measured skatole values in plasma were between 161 ng/dL and the detection limit or even below, respectively. According to Babol et al. [46] skatole concentrations of 1260 ng/dL in plasma correspond to skatole levels in adipose tissue above the threshold for perceptibility by consumers, which is assumed to be 250 ng/g adipose tissue. In the current study only one animal (1.85%) exceeded this threshold for skatole (by far) with 1000 ng/g fat. Contradictory to Babol et al. [46], this animal had a plasma skatole level of 388 ng/dL three days before slaughter and even less at day 15 (95.4 ng/dL) and day 1 (12.4 ng/dL), respectively. Additionally, in terms of androstenone levels in adipose tissue only 12.9% of the animals in the present study exceeded the threshold of 1000 ng/g which is less in comparison to the study by Kress et al. [47] in finishing boars (pietrain × hybrid) where 79% of the animals exceeded the threshold for androstenone levels and 6.25% the threshold for skatole in adipose tissue.

Since previous studies revealed that there may be a dependence between prevailing pH and skatole production [12] and additionally, biochar was shown to influence the pH in pig manure [48], the faecal pH was measured in the present study. Even though treatments did not differ in terms of pH_f_, positive correlations were found between pH_f_ and faecal I at day 1 and 15 but not at day 26. However, pH_f_ was significantly higher at day 26 than at day 1 and day 15, so this may have affected the correlation. Nevertheless, no correlations were found between pH_f_ and faecal S. According to Knarreborg et al. [49] the concentrations of skatole in faeces and blood are highly correlated within individual animals, whereas correlations between animals are less pronounced. In the present study, weak positive correlations were found between skatole in faeces and in plasma at day 1 and at day 15, but at day 26 the skatole concentrations in faeces and plasma only tended to correlate (*p* = 0.093). In addition, in agreement with Knarreborg et al. [49], the authors found that the indole levels in faeces did not correlate with corresponding levels in plasma. Although it is striking that T1 differed from T3 (and partly from T2) in terms of both skatole levels and performance parameters, no correlations between performance parameters and skatole contents could be found in the present study.

As previous studies in piglets with pure biochar did not lead to a reliable reduction in skatole and indole (data not published), a fat coating was applied to the biochar. This was supposed to hinder an unwanted loading of the biochar with nutrients from the chyme and was expected to be digested by endogenous enzymes during the passage through the small intestines so that the biochar would be free of fat and its pores unloaded when entering the hindgut. As the coated biochar has already been pretested on a small scale in piglets (data not shown), it was decided to test the coated biochar in finishing boars. Nevertheless, in the faeces of both feeding groups (CON and BCF), fat particles were macroscopically visible, giving an indication that the rapeseed fat had not been fully digested. Therefore, it is questionable as to what proportion of the biochar was effectively exposed in the large intestine of the pigs and could theoretically have bound to odour components. Therefore, in future studies, a more digestible fat should probably be used as the coating raw material. Similar to a previous study by Jen and Squires [31], which could not achieve a significant reduction in skatole levels when feeding 5% activated carbon to finishing boars, low skatole levels might also be considered an underlying cause in the present study. The use of coated biochar in field studies may be interesting since on the one hand much larger samples could be investigated and, on the other hand, under practical conditions, stress often occurs due to regrouping of animals prior to slaughter or (subclinical) infections, which may increase skatole formation [50].

Additionally, with a view to a possible use of the coated biochar in commercial pork production systems, effects on faecal properties and performance parameters were also investigated. The DM_f_ was higher when pigs were fed BCF, which became significant at day 15 (CON: 283 ^b^ ± 23.4; BCF: 301 ^a^ ± 29.2 g/kg) and day 26 (CON: 286 ^b^ ± 27.4; BCF: 305 ^a^ ± 31.7 g/kg). A former study conducted in piglets, already showed that adding two different biochars at a dietary level of 2% increased the DM_f_ by 9.31% (biochar 1) and 13.1% (biochar 2), respectively, compared to the control group [51]. Against the background that charcoal has been used for centuries as a household remedy for diarrhoea [35], these results are understandable. The antidiarrhoeal effect could be explained by the fact that charcoal was shown to bind bacterial pathogens [35]. However, the animals in the present study showed no clinical signs of diarrhoea. Therefore, the differences in DM_f_ between the feeding groups are presumably only marginally pronounced.

At the beginning of the study, there was no difference in BW in relation to the different treatments (BC0, BC2, BC4) and different trials (T1, T2, T3), while at day 28, the BW in T3 was significantly lower than in T1 (*p* = 0.021). With regard to ADWG (T1: 925 ^a^ ± 79.0; T2: 798 ^b^ ± 128; T3: 788 ^b^ ± 136 g/d) and FCR (T1: 0.394 ^a^ ± 0.020; T2: 0.332 ^b^ ± 0.049; T3: 0.334 ^b^ ± 0.058), there were clear differences between the trials (*p* = 0.001 and *p* < 0.001, respectively). In trial 1, the FCR was nearly the same for all treatments (BC0: 0.394, BC2: 0.392, BC4: 0.396), whereas in trials 2 and 3 (BC0: 0.307; BC2: 0.344; BC4: 348), it seemed that the administering biochar offered an advantage over the control diet. If the lower performance in trials T2 and T3 was related to subclinical infections, the biochar may have positively influenced the infection process.

## 5. Conclusions

Feeding biochar did not result in a measurable decrease skatole and indole concentrations either in faeces or plasma. However, concentrations in both matrices were in general quite low and only one animal exceeded the threshold for skatole content in adipose tissue. Nevertheless, feeding biochar does not have negative effects on the performance and boar taint levels of the animals.

## Figures and Tables

**Table 1:** Composition of the diets as percentage.

Feedstuffs	Variant 1	Variant 2
Barley	21.0	21.0
Wheat	15.0	20.0
Triticale	14.0	16.0
Rye	13.0	15.0
Wheat bran	13.0	8.0
Soybean meal *	5.0	6.0
Maize	5.0	5.0
Oat hulling bran	5.0	–
Wheat semolina bran	2.0	2.0
Maize gluten feed	2.0	2.0
Rapeseed meal	2.0	2.0
Premix **	3.0	3.0

* Soybean meal made from genetically modified soybeans. ** Additives (per kg feed); nutritional additives: variant 1: vitamin A (5100 IU), vitamin D/vitamin D3 (1525 IU), Vitamin E (41 mg), iron from iron-(II)-sulfate monohydrate (61 mg), copper from copper-(II)-sulfate pentahydrate (10 mg), manganese from manganese-(II)-oxide (41 mg), zinc from zincoxide (61 mg), iodine from calcium iodate anhydrous (2.0 mg), selenium from sodium selenite (0.36 mg); variant 2: vitamin A (5200 IU), vitamin D/vitamin D3 (1550 IU), Vitamin E (42 mg), iron from iron-(II)-sulfate monohydrate (63 mg), copper from copper-(II)-sulfate pentahydrate (10 mg), manganese from manganese-(II)-oxide (42 mg), zinc from zincoxide (63 mg), iodine from calcium iodate anhydrous (2.1 mg), selenium from sodium selenite (0.36 mg).

**Table 2:** Energy content and chemical composition of the experimental diets.

Item		CON ^1^	BCF ^2^
Metabolizable energy (ME) ^3^	MJ per kg diet	13.0	13.1
Organic matter	g/kg DM ^4^	951	952
Crude protein	g/kg DM	157	160
Ether extract	g/kg DM	56.9	52.1
Crude fibre	g/kg DM	56.6	52.0
Nitrogen-free extract (NfE) ^5^	g/kg DM	681	688
Calcium	g/kg DM	5.95	6.48
Phosphorus	g/kg DM	4.87	4.70
Magnesium	g/kg DM	2.00	1.94
Sodium	g/kg DM	2.36	2.21
Zinc	mg/kg DM	130	133
Iron	mg/kg DM	315	344
Selenium	mg/kg DM	0.676	0.835
Copper	mg/kg DM	27.9	29.8

^1^ CON, control feed. ^2^ BCF, biochar containing feed. ^3^ Metabolizable energy (ME) calculated from the specific raw nutrient content. ^4^ DM, dry matter. ^5^ Nitrogen-free extract (NfE) = dry matter—(crude ash + crude protein + ether extract + crude fibre).

**Table 3:** Faecal dry matter content (DM_f_, mean ± SD) and faecal pH (pH_f_, mean ± SD) in dependence on feed affiliation ^1^ (CON, BCF).

Faecal Properties	Feed Affiliation				*p*
	CON ^2^	n	BCF ^3^	n	
DM_f_ day 1 (g/kg)	274 ± 24.9	52	--	0	--
DM_f_ day 8 (g/kg)	279 ± 24.1	35	287 ± 29.9	18	0.295
DM_f_ day 15 (g/kg)	283 ^b^ ± 23.4	36	301 ^a^ ± 29.2	18	0.015
DM_f_ day 22 (g/kg)	293 ± 23.6	18	296 ± 24.7	35	0.711
DM_f_ day 26 (g/kg)	286 ^b^ ± 27.4	18	305 ^a^ ± 31.7	36	0.034
pH_f_ day 1	6.77 ± 0.361	53	--	0	--
pH_f_ day 8	6.77 ± 0.271	36	6.80 ± 0.395	18	0.719
pH_f_ day 15	6.83 ± 0.304	35	6.76 ± 0.268	18	0.463
pH_f_ day 22	6.78 ± 0.257	18	6.82 ± 0.359	36	0.680
pH_f_ day 26	6.98 ± 0.255	18	7.03 ± 0.338	35	0.614

^1^ The diet fed the previous day is considered as feed affiliation. ^2^ CON, control feed. ^3^ BCF, biochar containing feed. ^a,b^ Superscripts indicate significance. Means with common superscripts are not significantly different.

**Table 4:** Bodyweight (BW, mean ± SD), average daily weight gain (ADWG, mean ± SD), gain-to-feed-ratio ^1^ (G:F, mean ± SD) in dependence on treatment (BC0, BC2, BC4) and trial (T1, T2, T3).

Performance Parameter	Treatment			*p*	Trial			*p*
	BC0	BC2	BC4		T1	T2	T3	
BW day 1 (kg)	97.3 ± 5.56	96.7 ± 7.47	97.7 ± 7.76	0.927	97.5 ± 8.58	98.6 ± 6.57	95.6 ± 5.08	0.471
BW day 28 (kg)	119 ± 5.10	119 ± 7.01	120 ± 6.69	0.767	122 ^a^ ± 7.37	120 ^ab^ ± 6.05	117 ^b^ ± 4.10	0.063
ADWG (g/d)	806 ± 159	837 ± 126	865 ± 102 ^2^	0.262	925 ^a^ ± 79.0 ^2^	798 ^b^ ± 128	788 ^b^ ± 136	0.001
G:F (kg/kg)	0.336 ± 0.067	0.360 ± 0.047	0.362 ± 0.041 ^2^	0.134	0.387 ^a^ ± 0.034 ^2^	0.332 ^b^ ± 0.049	0.334 ^b^ ± 0.058	<0.001

^1^ Gain-to-feed-ratio calculated as gain per feed. ^2^ One outlier was removed from the statistic (*n* = 17). ^a,b^ Superscripts indicate significance. Means in the same row with common superscripts are not significantly different.

## Data Availability

The data presented in this study are available in this manuscript and the supplementary material.

## Supplementary Materials

The following are available online at https://www.mdpi.com/2076-2615/11/3/760/s1, Table S1: Faecal concentrations of skatole (S, mean ± SD) indole (I, mean ± SD) and sum of skatole and indole (S + I, mean ± SD) given as µg/g DM in dependence on treatment and trial (*n* = 18, *N* = 54), Table S2: Changes in the faecal concentrations of skatole (S), indole (I) and sum of skatole and indole (S + I) between measurements at day 1 and day 15 (day 1–day 15), day 15 and day 26 (day 15–day 26) as well as day 1 and day 26 (day 1–day 26) given as µg/g DM in dependence on treatment and trial (*n* = 18, *N* = 54), Table S3: Plasma concentrations of skatole (S, mean ± SD) indole (I, mean ± SD) and sum of skatole and indole (S + I, mean ± SD) given as ng/dL in dependence on treatment and trial (*n* = 18, *N* = 54), Table S4: Changes in the plasma concentrations of skatole (S), indole (I) and sum of skatole and indole (S + I) between measurements at day 1 and day 15 (d15–d1), day 15 and day 26 (d26–d15) as well as day 1 and day 26 (d26–d1) given as ng/dL in dependence on treatment and trial (*n* = 18, *N* = 54), Table S5: Correlation coefficients between boar taint compounds, faecal properties and performance parameters.
