# Supplementary material for: Evaluation of Coated Biochar as an Intestinal Binding Agent for Skatole and Indole in Male Intact Finishing Pigs
Source: Animals (Basel). 2021 Mar 10;11(3):760. doi: 10.3390/ani11030760 (PMC7998114; doi:10.3390/ani11030760)
Supplement: Supplementary file 1 [file animals-11-00760-s001.pdf]

Supplementary Materials

Table S1. Faecal concentrations of skatole (S, mean ± SD) indole (I, mean ± SD) and sum of skatole and indole (S+I, mean ± SD) given as µg/g DM in dependence on treatment and trial (n = 18, N = 54).

	Treatment				Trial			
	**BC0**	**BC2**	**BC4**	***p***	**T1**	**T2**	**T3**	***p***
S d1	135 ± 122	163 ± 118	78.6 ± 60.6	0.064	139 ± 126	126 ± 95.3	112 ± 105	0.742
S d15	207 ± 90.2	189 ± 123	202 ± 119	0.823	288^{a} ± 130	166^{b} ± 63.6	144^{b} ± 62.4	< 0.001
S d26	138 ± 69.2	128 ± 64.7	116 ± 68.9	0.574	155^{a} ± 75.9	130^{ab} ± 59.3	97.5^{b} ± 54.2	0.032
I d1	57.3 ± 34.4	64.0 ± 35.4	53.5 ± 28.7	0.577	70.1^{a} ± 36.2	63.0^{a} ± 27.1	41.7^{b} ± 28.5	0.020
I d15	69.0 ± 33.8	76.6 ± 42.0	52.5 ± 36.0	0.146	80.9 ± 49.4	53.7 ± 27.9	63.6 ± 30.1	0.093
I d26	54.8 ± 23.9	38.9 ± 25.0	39.8 ± 21.8	0.076	47.7 ± 22.8	36.0 ± 21.4	49.7 ± 27.4	0.168
S+I d1	192^{ab} ± 119	227^{a} ± 112	131^{b} ± 76.9	0.031	209 ± 121	188 ± 97.4	152 ± 108	0.273
S+I d15	276 ± 83.1	265 ± 117	255 ± 147	0.785	369^{a} ± 131	220^{b} ± 72.4	207^{b} ± 54.4	< 0.001
S+I d26	193 ± 59.1	167 ± 65.8	156 ± 68.9	0.185	203 ± 77.4	166 ± 53.9	147 ± 52.4	0.030

$^{a, b}$ Superscripts indicate significance. Means in the same row with common superscripts are not significantly different.

Table S2. Differences[1] in the faecal concentrations of skatole (S), indole (I) and sum of skatole and indole (S+I) between measurements at day 1 and day 15 (d15 - d1), day 15 and day 26 (d26 - d15) as well as day 1 and day 26 (d26 - d1) given as µg/g DM in dependence on treatment and trial (n = 18, N = 54).

	Treatment				Trial			
	**BC0**	**BC2**	**BC4**	***p***	**T1**	**T2**	**T3**	***p***
S d15 - d1	72.0^{ab} ± 92.7	25.4^{b} ± 162	124^{a} ± 116	0.047	149^{a} ± 153	40.0^{b} ± 85.9	32.2^{b} ± 116	0.006
S d26 - d15	-68.5 ± 91.7	-60.4 ± 110	-86.3 ± 122	0.747	-133^{b} ± 154	-35.7^{a} ± 48.2	-46.4^{a} ± 60.5	0.013
S d26 - d1	3.50 ± 111	-35.0 ± 102	37.4 ± 85.6	0.107	15.8 ± 124	4.36 ± 83.8	-14.2 ± 100	0.667
I d15 - d1	11.7 ± 44.3	12.6 ± 50.7	-0.96 ± 32.8	0.582	10.7 ± 48.1	-9.36 ± 34.7	22.0 ± 41.1	0.102
I d26 - d15	-14.2 ± 27.8	-37.7 ± 42.4	-12.8 ± 40.5	0.098	-33.1 ± 50.0	-17.6 ± 29.2	-13.9 ± 32.4	0.283
I d26 - d1	-2.49 ± 35.5	-25.1 ± 38.7	-13.7 ± 32.1	0.130	-22.4^{b} ± 41.4	-27.0^{b} ± 30.6	8.06^{a} ± 25.4	0.005
S+I d15 - d1	84.3 ± 109	38.2 ± 144	123 ± 134	0.107	160^{a} ± 157	31.2^{b} ± 88.3	55.1^{b} ± 110	0.005
S+I d26 - d15	-82.7 ± 87.7	-98.0 ± 105	-99.4 ± 153	0.883	-166^{b} ± 166	-53.2^{a} ± 52.6	-60.3^{a} ± 57.8	0.007
S+I d26 - d1	1.57 ± 116	-59.7 ± 98.8	24.4 ± 101	0.065	-6.62 ± 138	-22.0 ± 90.0	-5.20 ± 101	0.521

[1] Differences calculated from dependent samples of the raw data and presented as mean ± SD. A negative value indicates that mean values of the latter measurement are (numerically) lower.

Table S3. Plasma concentrations of skatole (S, mean ± SD) indole (I, mean ± SD) and sum of skatole and indole (S+I, mean ± SD) given as ng/dL in dependence on treatment and trial (n = 18, N = 54).

	Treatment				Trial			
	**BC0**	**BC2**	**BC4**	***p***	**T1**	**T2**	**T3**	***p***
S d1	12.7 ± 11.8	29.1 ± 49.5	8.94 ± 9.67	0.529	8.72 ± 10.4	19.0 ± 34.4	23.1 ± 38.9	0.203
S d15	28.0 ± 30.5	22.6 ± 27.0	19.5 ± 19.6	0.442	23.7 ± 23.4	23.2 ± 25.0	23.2 ± 30.2	0.735
S d26	31.9 ± 89.2	21.6 ± 20.2	13.3 ± 13.2	0.276	11.0^{b} ± 7.79	43.1^{a} ± 87.5	12.7^{b} ± 18.0	0.010
I d1	15.4 ± 13.9	29.0 ± 52.7	13.5 ± 10.0	0.683	12.9 ± 4.73	16.6 ± 19.0	28.4 ± 52.0	0.759
I d15	24.6 ± 23.8	28.8 ± 41.7	22.8 ± 21.6	0.971	17.6^{b} ± 6.82	17.7^{b} ± 5.90	40.9^{a} ± 48.3	0.024
I d26	26.2 ± 18.7	22.8 ± 18.6	19.8 ± 11.3	0.503	20.8 ± 7.48	21.3 ± 16.8	26.7 ± 21.9	0.391
S+I d1	27.2 ± 21.3	56.9 ± 50.5	20.7 ± 17.5	0.549	19.7 ± 12.0	34.4 ± 53.0	50.6 ± 88.7	0.874

S+I d15	52.6 ± 52.1	50.5 ± 62.5	41.2 ± 30.9	0.653	40.5 ± 27.9	40.0 ± 30.2	63.7 ± 74.7	0.611
S+I d26	57.1 ± 104	42.8 ± 36.9	32.0 ± 20.3	0.501	30.2 ± 13.9	63.3 ± 103	38.3 ± 37.6	0.237

Table S4. Differences[1] in the plasma concentrations of skatole (S), indole (I) and sum of skatole and indole (S+I) between measurements at day 1 and day 15 (d15-d1), day 15 and day 26 (d26-d15) as well as day 1 and day 26 (d26-d1) given as ng/dL in dependence on treatment and trial (n = 18, N = 54).

	Treatment				Trial			
	**BC0**	**BC2**	**BC4**	***p***	**T1**	**T2**	**T3**	***p***
S d15 - d1	15.3 ± 29.0	-6.52 ± 44.5	10.6 ± 14.1	0.292	15.0 ± 24.4	4.23 ± 36.9	0.11 ± 34.9	0.203
S d26 - d15	3.87 ± 77.0	-0.97 ± 22.6	-6.25 ± 16.4	0.070	-12.8[b] ± 23.2	19.9[a] ± 69.4	-10.5[b] ± 26.5	0.037
S d26 - d1	19.2 ± 90.0	-7.49 ± 39.3	4.33 ± 10.9	0.798	2.26[a] ± 12.9	24.2[a] ± 93.3	-10.4[b] ± 25.1	0.004
I d15 - d1	9.23 ± 12.8	-0.28 ± 20.9	9.35 ± 19.2	0.395	4.75 ± 6.83	1.07 ± 18.8	12.5 ± 23.7	0.388
I d26 - d15	1.58 ± 18.1	-5.90 ± 24.8	-3.02 ± 16.1	0.381	3.18[a] ± 9.62	3.65[a] ± 12.5	-14.2[b] ± 27.7	0.013
I d26 - d1	10.8 ± 15.0	-6.18 ± 36.8	6.32 ± 9.08	0.378	7.92 ± 8.02	4.72 ± 23.5	-1.69 ± 34.1	0.559
S+I d15 - d1	25.4 ± 39.6	-6.44 ± 62.4	20.5 ± 23.4	0.239	20.8 ± 30.1	5.57 ± 52.9	13.0 ± 53.0	0.957
S+I d26 - d15	4.56 ± 92.1	-7.67 ± 35.0	-9.18 ± 24.1	0.696	-10.3[ab] ± 28.0	23.3[a] ± 79.6	-25.3[b] ± 44.5	0.008
S+I d26 - d1	29.9 ± 104	-14.1 ± 71.1	11.3 ± 16.0	0.899	10.5 ± 15.5	28.9 ± 113	-12.3 ± 55.4	0.064

[1] Differences calculated from the raw data and presented as mean value ± SD. A negative value indicates that mean values of the latter measurement are (numerically) lower.

Table S5. Correlation coefficients between boar taint compounds, faecal properties and performance parameters.

**Correlated paramaters**	**Treatment**	**n**	**Spearman's rank correlation coefficient**	**p-value**
	All	54	0.424	0.001
Skatole faeces d1	BC0	18	0.653	0.003
Skatole plasma d1	BC2	18	0.500	0.035
	BC4	18	0.119	0.637
	All	54	0.373	0.005
Skatole faeces d15	BC0	18	0.291	0.241
Skatole plasma d15	BC2	18	0.484	0.042
	BC4	18	0.331	0.180
	All	54	0.231	0.093
Skatole faeces d26	BC0	18	0.482	0.043
Skatole plasma d26	BC2	18	0.109	0.666
	BC4	18	0.114	0.653
	All	54	0.042	0.762
Indole faeces d1	BC0	18	0.385	0.115
Indole plasma d1	BC2	18	-0.260	0.298
	BC4	18	0.076	0.766
	All	54	0.060	0.664
Indole faeces d15	BC0	18	-0.201	0.423
Indole plasma d15	BC2	18	0.174	0.488
	BC4	18	0.001	0.996

Indole faeces d26<br>Indole plasma d26	All	54	0.077	0.582
	BC0	18	-0.059	0.817
	BC2	18	0.011	0.964
	BC4	18	0.098	0.699
Skatole faeces d1<br>Skatole faeces d26	All	54	0.355	0.008
	BC0[1]	18	0.434	0.072
	BC2	18	0.459	0.055
	BC4	18	0.078	0.757
Indole faeces d1<br>Indole faeces d26	All	54	0.205	0.136
	BC0	18	0.267	0.284
	BC2	18	0.148	0.559
	BC4	18	0.300	0.226
Skatole plasma d1<br>Skatole plasma d26	All	54	0.356	0.008
	BC0	18	0.161	0.523
	BC2	18	0.692	0.002
	BC4	18	0.160	0.527
Indole plasma d1<br>Indole plasma d26	All	54	0.533	< 0.0001
	BC0	18	0.689	0.002
	BC2	18	0.281	0.260
	BC4	18	0.522	0.026
Skatole plasma d26<br>Skatole back fat	All	54	0.706	< 0.0001
	BC0	18	0.843	< 0.0001
	BC2	18	0.545	0.019
	BC4	18	0.484	0.042
Indole plasma d26<br>Indole back fat	All	54	0.554	< 0.0001
	BC0	18	0.535	0.022
	BC2	18	0.488	0.040
	BC4	18	0.529	0.024
Skatole back fat<br>Indole back fat	All	54	0.090	0.518
	BC0	18	0.017	0.948
	BC2	18	0.266	0.367
	BC4	18	-0.015	0.951
Skatole back fat<br>Androstenone back fat	All	54	-0.049	0.724
	BC0	18	-0.155	0.538
	BC2	18	-0.123	0.627
	BC4	18	-0.070	0.782
Indole back fat<br>Androstenone back fat	All	54	0.345	0.011
	BC0	18	0.410	0.091
	BC2	18	0.430	0.075
	BC4	18	0.097	0.701
Skatole faeces day 1<br>Faecal pH day 1	All	53	-0.167	0.276
	BC0	18	-0.306	0.217
	BC2[1]	17	0.230	0.374
	BC4[1]	18	-0.155	0.538

Skatole faeces day 15<br>Faecal pH day 15	All	53	-0.075	0.594
	BC0[1]	17	-0.242	0.350
	BC2	18	-0.317	0.200
	BC4	18	0.293	0.238
Skatole faeces day 26<br>Faecal pH day 26	All	53	0.107	0.445
	BC0[1]	18	0.001	0.995
	BC2[1]	18	0.076	0.764
	BC4	17	0.288	0.262
Indole faeces day 1<br>Faecal pH day 1	All	53	0.378	0.005
	BC0	18	0.585	0.011
	BC2[1]	17	0.625	0.007
	BC4[1]	18	-0.049	0.847
Indole faeces day 15<br>Faecal pH day 15	All	53	0.498	0.0001
	BC0[1]	17	0.627	0.007
	BC2[1]	18	0.486	0.041
	BC4	18	0.379	0.121
Indole faeces day 26<br>Faecal pH day 26	All	53	0.147	0.294
	BC0[1]	18	0.249	0.319
	BC2	18	0.205	0.414
	BC4	17	0.134	0.609
Skatole faeces day 1<br>BW day 1	All	54	0.048	0.728
Skatole faeces day 15<br>BW day 15	All	54	0.013	0.927
Skatole faeces day 26<br>BW day 26	All	53	0.109	0.435
Skatole plasma day 1<br>BW day 1	All	54	-0.091	0.513
Skatole plasma day 15<br>BW day 15	All	54	-0.154	0.267
Skatole plasma day 26<br>BW day 26	All	53	0.154	0.272
Skatole faeces (mean)[2]<br>ADWG	All	53	0.197	0.157
Skatole faeces (mean)<br>FCR	All	53	0.187	0.181
Indole faeces (mean)<br>ADWG	All	53	0.104	0.461
Indole faeces (mean)<br>FCR	All	53	0.114	0.418
Skatole plasma (mean)[2]<br>ADWG	All	53	-0.221	0.112

Skatole plasma (mean) FCR	All	53	-0.245	0.077
Indole faeces (mean) ADWG	All	53	-0.083	0.553
Indole faeces (mean) FCR	All	53	-0.064	0.650

[1] Pearson's correlation coefficient. [2] Mean value of measurements at day 1, 15 and 26.
